# Deformed wing virus of honey bees is inactivated by cold plasma ionized hydrogen peroxide

**DOI:** 10.3389/finsc.2023.1216291

**Published:** 2023-08-02

**Authors:** Steven C. Cook, Eugene V. Ryabov, Christian Becker, Curtis W. Rogers, Francisco Posada-Florez, Jay D. Evans, Yan Ping Chen

**Affiliations:** ^1^ United States Department of Agriculture - Agricultural Research (USDA-ARS) Service, Bee Research Laboratory, Beltsville, MD, United States; ^2^ Department of Entomology, University of Maryland, College Park, MD, United States; ^3^ Arkema, Inc., King of Prussia, PA, United States

**Keywords:** Apis mellifera, DWV, IHP, infectivity, Varroa destructor

## Abstract

Deformed wing virus (DWV) is a widespread pathogen of *Apis mellifera* honey bees, and is considered a major causative factor for the collapse of infected honey bee colonies. DWV can be horizontally transmitted among bees through various oral routes, including via food sharing and by interactions of bees with viral-contaminated solid hive substrates. Cold plasma ionized hydrogen peroxide (iHP) is used extensively by the food production, processing and medical industries to clean surfaces of microbial contaminants. In this study, we investigated the use of iHP to inactivate DWV particles *in situ* on a solid substrate. iHP-treated DWV sources were ~10^5^-fold less infectious when injected into naïve honey bee pupae compared to DWV receiving no iHP treatment, matching injected controls containing no DWV. iHP treatment also greatly reduced the incidence of overt DWV infections (i.e., pupae having >10^9^ copies of DWV). The level of DWV inactivation achieved with iHP treatment was higher than other means of viral inactivation such as gamma irradiation, and iHP treatment is likely simpler and safer. Treatment of DWV contaminated hive substrates with iHP, even with honey bees present, may be an effective way to decrease the impacts of DWV infection on honey bees.

## Introduction

Deformed wing virus (DWV) is a positive-stranded RNA virus (*Iflaviradae*) that infects several hymenopteran taxa including bees, wasps, and ants (1–3). A growing body of evidence suggests that spread of the mite *Varroa destructor* (hereafter, Varroa) has greatly increased pathogenicity of DWV (4–6), making it the most important viral pathogen of the Western honey bee, *Apis mellifera* (7). Symptoms of overt DWV infections of adult honey bees include damaged appendages, particularly stubby, useless wings, shortened, rounded abdomens, and miscoloring and paralysis of the legs and wings. These symptoms are strongly correlated with elevated DWV levels (*i.e*., genome equivalents (GE)) (8). DWV infections can reduce the lifespan of covertly infected adult honey bees (9, 10).

In the absence of mites, the virus persists in bee populations as a covert infection, transmitted horizontally (11) via several routes, including orally among adults through trophallaxis (12) and between adults and immature bees *via* hypopharyngeal gland secretions (13) and brood food (14) fed to larvae, and possibly through a fecal–oral route of transmission between adult bees (15, 16). Workers may also be infected through feeding on DWV-contaminated hive products, such as pollen (17) and honey (18), or by cannibalizing infected pupae (12). Adult honey bees clean hive substrates, including the meconium and other contents remaining in pupal cells of newly-eclosed adults, possibly becoming exposed to latent DWV on interior surfaces of hives (19, 20). While little work has been conducted to investigate this latter route, honey bees were found to become infected by interacting with DWV-contaminated beeswax (18).

Persistence of virus infectivity in the environment can vary; Enveloped viruses, such as influenza and coronaviruses, are considered less stable than non-enveloped viruses, such as norovirus (21). There is little information on the persistence of DWV infectivity outside their hosts, but considering that DWV virions are similar to those of other Picorna-like viruses, it is very likely that environmental DWV contamination plays a significant role in circulation of DWV. Indeed, infection can occur in adult bees contacting contaminated beeswax (18). Thus, deactivating latent DWV and other honey bee viral pathogens on hive substrates may reduce incidence of infection for resident bees. Treatment of surfaces with iHP, or ionized hydrogen peroxide is widespread in hospitals and other spaces where decontamination from microbial pathogens is required (22). Specifically, iHP treatment has been shown to be effective against the coronavirus (23, 24), as well as other microbial pathogens (25). Hydrogen peroxide is a potent oxidizer and is believed to inactivate viruses through oxidation of protein side chains (26) and nucleotides (27). In this study, we explore the use of iHP to reduce the infectivity of DWV to naïve honey bee pupae.

## Materials and methods

### Source of honey bee deformed wing virus

Clone-derived DWV-A isolate Maryland/2015/422 (GenBank: MG831202) (28) was used in this experiment. The virus was propagated in honeybee pupae injected with infectious *in vitro* RNA transcripts (28). For preparation of extracts containing infectious DWV virus particles, individual transcript-injected pupae were homogenized with 2 mL of PBS at 3 days post-injection, subjected to three freeze–thaw cycles, clarified by centrifugation at 3000g for 5 min and filtered through 0.22 μm nylon filter (Millipore). The DWV concentration in these extracts was quantified by qRT-PCR. The original DWV-A extract used in this study had 10^8^ genome equivalents (GE) of DWV per microliter. The infectivity of the clone-derived DWV extracts was confirmed by the presence of high levels of DWV infections (10^11^ to 10^12^ GE/pupae) in the pupae injected with 10^6^ GE in 10 μL of PBS 3 days prior. The extracts were stored at -80°C prior to use. The identity of clonal DWV in the extract with its respective parental cDNA clone was confirmed by sequencing of RT-PCR fragments (29).

### Contamination of surfaces with DWV particles

**Figure 1:**
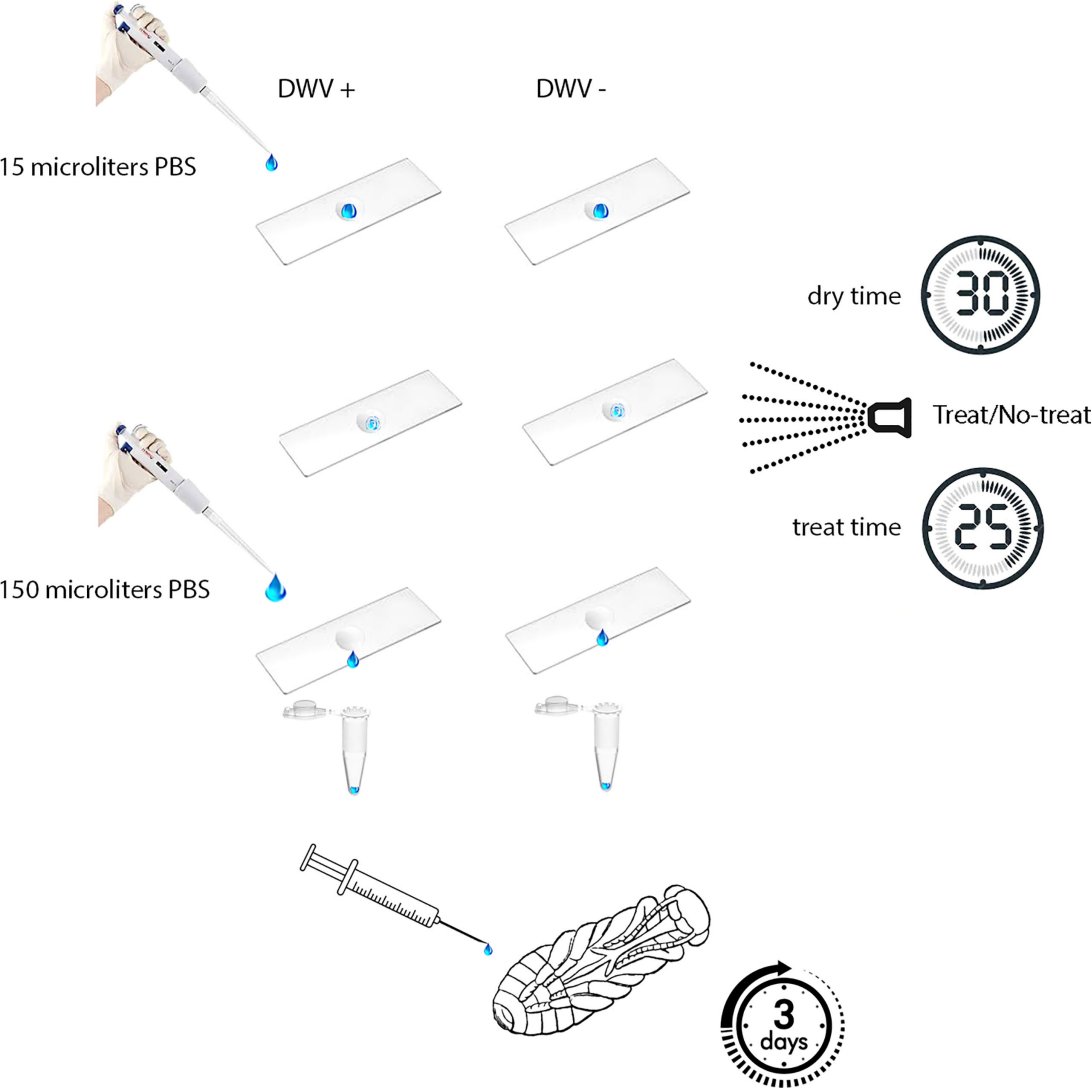
Experimental design. Aliquots of PBS either with or without DWV were placed on a non-porous surface, allowed to dry, then either treated or untreated with ionized hydrogen peroxide (iHP). Aliquots were recovered from surfaces and then injected in naïve honey bee pupae. DWV infection progressed for three days prior to pupae being analyzed for their DWV content (*i.e.*, number of genome equivalents).

To assess the effect of iHP treatment on DWV infectivity, DWV were placed on a non-porous surface were exposed to iHP treatment, then tested for their ability to infect honey bee pupae (**Figure 1**). For this, 15 μL of DWV extract in PBS at 10^8^ DWV particles per 1 μL were placed on a thin, 2 cm x 5 cm rectangular steel sheet. Prior to application of the virus suspension, the metal surface was sterilized with 100% ethanol, washed with sterile water and air-dried. As a control, 15 μL of PBS containing no virus extracts were also pipetted onto a steel sheet. Each 15 μL aliquot was evenly spread to cover approximately 2 cm^2^; this area was delineated with an ultra-fine marker prior to placing the liquid aliquots. Aliquots were air-dried at room temperature for 30 minutes. The contents of the aliquots were recovered from the surfaces by washing the marked areas with 150 μL of PBS; resulting in virus suspensions that were assumed to have a concentration of 10^7^ DWV particles per 1 μL. The viral suspensions and control solutions were stored at -80 °C prior to testing for infectivity.

### Treatment with ionized hydrogen peroxide

iHP mist exposure occurred in a clean, sealed 14 ft by 9 ft by 9 ft (~4.3 m x ~2.7 m x ~2.7 m) rectangular environmental chamber located at the USDA-ARS Bee Research Laboratory, Beltsville, MD. iHP mist was introduced into the treatment chamber using SteraMist application technology (from TOMI™ Environmental Solutions). Binary Ionization Technology (BIT) Solution (TOMI Environmental Solutions, Inc.) was applied at a dose rate of 0.5 mL/ft^3^ (~0.03 m^3^). In the application process, first the BIT Solution passed through a nozzle that generated an ultra-fine 0.05 – 3-micron particle mist (25). Subsequently, the mist was ionized by cold plasma generated between two pin electrodes. Two applicators were placed in opposite corners of the room. The room was sterilized prior to treating the inoculated steel plates by applying 567 mL of BIT Solution at a rate of 25 mL/min per applicator, resulting in the dose concentration of 0.5 mL per cubic foot for the treated area. The application time for the dose was 10 min and was followed by a contact time of 15 min (**Figure 1**). Following the application and contact time, remaining iHP mist was removed from the chamber through an inline exhaust fan to outside until the hydrogen peroxide ppm levels in the chamber were measured at <1 ppm (Draeger, Inc. USA) (~15 mins). This aeration process was shortened by the natural breakdown of the hydrogen peroxide to water and oxygen. When the room was cleared of residual hydrogen peroxide, steel plates were placed with the inoculated area facing up on a cart previously placed in the room and sterilized, and the room was again treated as above. Following aeration, the steel plates were removed from the room and returned to the laboratory where both the virus and PBS control aliquots were recovered from steel plates as described above. The presence of hydrogen peroxide mist in the room after the application dose was applied and confirmed by iodine indicator strips (LaMotte, Maryland, USA), which change color from yellow to various blue shades based on the level of hydrogen peroxide present in the room.

Blank treatments were conducted by running sterile water through the SteraMist application equipment and removing the electrodes to prevent the plasma arc from occurring, so ionization did not occur.

### Assessment of treated DWV extract infectivity

The recovered virus suspensions and PBS controls, both treated and untreated, were diluted prior to being injected into pupae for tests of viral infectivity. Pink-eye stage honey bee pupae, with no exposure to *Varroa* mite feeding, were extracted from a brood frame with low *Varroa* infestation (less than 1 mite-infested pupae per 100). Pupae were intra-abdominally injected into the hemolymph to introduce 10^5^ and 10^6^ GE of surface-recovered DWV, H_2_O_2_-treated or control untreated, in 10 μL of PBS or buffer control (PBS) as described previously (5, 30). The injected pupae were incubated at +33° C, 75% relative humidity (31), for 3 days prior to RNA extraction (**Figure 1**). DWV RNA copy numbers were quantified in 8 individual pupae per treatment group by qRT-PCR (28). In brief, total RNA was extracted using Trizol (Ambion) method according to the manufacturer’s instructions. Then, cDNA was produced using Superscript II reverse transcriptase (Invitrogen) and random hexanucleotide primers according to manufacturer’s instructions. The cDNA samples were used to determine DWV copy numbers by quantitative PCR with the oligonucleotide primers 5’-GAGATCGAAGCGCATGAACA-3’ and 5’-TGAATTCAGTGTCGCCCATA-3’, targeting the region 6497 to 6626 in the DWV genome using SsoAdvanced Universal SYBR Green Supermix (Bio-Rad, Hercules, CA, USA). A series of 10-fold dilutions of the full-length cDNA clone of DWV, pDWV-422 (28) was used to establish a standard curve by plotting Ct values against the log-transformed concentrations of DWV cDNA. The Ct values showed linearity (r^2 ^= 0.998) and gave an amplification efficiency of 1.95.

### Statistical analyses

Data for the log_10_-transformed qPCR thresholds (indicating DWV loads) were not normally distributed, thus these data were analyzed using a Wilcoxon test. A Wilcoxon signed ranks test for multiple comparisons was used as a *post-hoc* test. This test generates Z-scores which are compared to a critical statistic, *W* (32). For the eight honey bees per treatment, those having greater than 10^9^ viral copies were contrasted with those having lower virus levels using a Chi-square test. For the latter, a contingency test was used to distinguish between-group differences.

## Results

**Figure 2:**
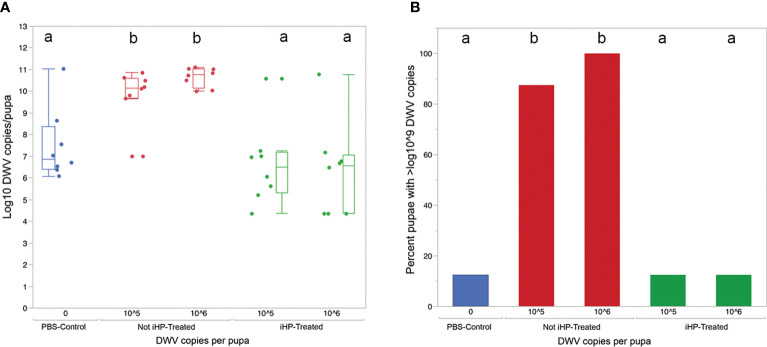
**(A)** Boxplot shows development of virus infection (log_10_ copy number of DWV) in honey bee pupae 3 days after injection with PBS (Control), 10^5^ and 10^6^ copies of DWV recovered from the surfaces and for the latter, either untreated (Not-iHP-treated) or treated with ionized hydrogen peroxide for 30 minutes (iHP-treated). Dots represent virus levels in individual pupae. Different ded letters above boxes indicate significantly different groups (p < 0.05, pairwise Wilcoxon test). **(B)** Percent of honey bee pupae having greater than 10^9^ GE of DWV.

The development of DWV infection in honey bees injected with the DWV inocula treated for 15 minutes with ionized hydrogen peroxide (iHP), for both the 10^5^ or 10^6^ GE doses, were significantly reduced compared to the honey bees injected with the untreated (*X^2 ^= *19.77; P < 0.0006). The PBS-injected (Control) pupae had similar levels of DWV GE to iHP-treated samples for both inoculations (all *P* > 0.05), while DWV levels were significantly elevated in non-treated samples compared to PBS-injected samples and both of the iHP-treated samples (all *P* < 0.05) (**Table 1**). Pupae injected with inocula containing either 10^5^ or 10^6^ copies of DWV that were untreated with iHP showed DWV titers elevated up to ~10^11^ copies of DWV per bee after 3 days, while pupae injected with iHP-treated DWV inoculates had the level of DWV statistically indistinguishable from those in the control, PBS-injected bees, ~10^3^-10^4^ time lower (**Figure 2A**). High DWV levels characteristic of overt infections (*i.e*., > 10^9^ copies/bee) were observed only in 1 of 8 (12.5%) of honey bees injected with either PBS or hydrogen peroxide-treated inocula, regardless of the DWV dose, while 7 of 8 (87.5%) and (8 of 8) 100% of honey bees injected with 10^5^ and 10^6^ viral copies, respectively, showed high DWV loads, 10^10^ - 10^11^, typical for overt DWV infection (**Figure 2B**). Such reduction of DWV infectivity after iHP treatment was statistically significant, for the 10^6^ GE inoculum, Fisher exact probability test, P = 0.0014).

**Table 1:** Statistical results from the Wilcoxon signed rank test for multiple comparisons of the log_10_ -transformed DWV GE quantified by RT-qPCR in honey bee pupae after three days post-injection with PBS having either 10^5^ or 10^6^ DWV copies of DWV or without DWV (Control).

Level	- Level	Score Mean Difference	Std Err Difference	Z-Score	p-Value	Hodges- Lehmann	Lower CL	Upper CL
Not treated 10^6^	Not-treated 10^5^	4.375	2.380	1.837	0.066	0.531	-0.116	1.297
iHP treated 10^6^	iHP treated 10^5^	-1.000	2.362	-0.423	0.672	-0.254	-2.652	1.964
iHP treated 10^6^	PBS Control	-2.625	2.373	-1.105	0.268	-1.270	-3.200	0.593
iHP treated 10^5^	PBS Control	-2.875	2.380	-1.207	0.227	-0.813	-2.683	0.709
PBS Control	Not treated 10^5^	-5.125	2.380	-2.152	0.031	-3.089	-3.951	-0.287
iHP treated 10^5^	Not treated 10^5^	-5.875	2.380	-2.467	0.013	-3.606	-5.000	-0.938
iHP treated 10^6^	Not treated 10^5^	-5.875	2.373	-2.475	0.013	-3.657	-5.842	-0.516
PBS Control	Not treated 10^6^	-6.375	2.380	-2.678	0.007	-3.642	-4.461	-2.085
iHP treated 10^6^	Not treated 10^6^	-6.875	2.373	-2.896	0.003	-4.297	-6.485	-3.264
iHP treated 10^5^	Not treated 10^6^	-7.125	2.380	-2.993	0.002	-4.089	-5.626	-3.032

Z-scores were compared against a critical W, which was 1.96 for this test.

## Discussion

Deformed wing virus (DWV) negatively affects honeybee health and can lead to colony losses (33, 34). Horizontal transmission of DWV among honey bees can occur via oral routes (12–16, 18), and DWV on hive surfaces could act as a reservoir for the virus. Research has demonstrated that overt DWV infections in honey bees can be caused by interactions between adult honey bees and hive substrates (e.g., wax) (18), and 60% - 80% of beeswax from brood frames was positive for a number of honey bee pathogenic viruses, including DWV (20). Virus picked up on body surfaces and ingested from fastidious hygiene of worker honey could be enough to cause overt infections; oral exposure of bumblebees to a single dose of 0.5 x 10^7^ viral particles can cause overt infections (35). Here, we show that treatment of DWV-contaminated surfaces with iHP significantly reduces both the infectivity of the virus to honey bee pupae (10^4^-10^5^ reduction) and incidence of overt infections (> 10^9^ GE of virus) with DWV in these hosts injected with iHP-treated DWV inoculates.

Past efforts to inactivate viral and other honeybee pathogens have employed a number of different disinfecting agents, including ozone, ethylene oxide, and gamma irradiation (20, 35–38). Focusing on viral inactivation, the ~10^5^-fold reduction in viral infectivity from iHP treatment seen in our study is similar to that shown for gamma irradiation. Irradiated honey bee-collected pollen containing an inoculum (10^-1^ to 10^-5^ dilution) of the honey bee virus, Israeli acute paralysis virus (IAPV), resulted in a 1000-fold reduction of the infectivity of this virus to bumblebees (35), and reduced viability of other honey bee viruses (37), while inoculate solutions containing DWV exposed to gamma radiation for nearly 10 hours, then subsequently injected into honey bee pupae, showed 10^4^ - 10^5^-fold reduction in the infectivity of the virus (38). Treatment of pathogen-laden corbicular pollen with ozone had limited effect on contaminating microbes (37). Ethylene oxide treatment of hive substrates successfully inactivates bacterial and microsporidian honey bee pathogens, but this method may not be as effective against viral pathogens (36).

Consideration of using the different available decontaminating treatments for inactivating honey bee microbial pathogens from hive surfaces should examine factors such as the treatment’s efficacy against a broad range of microbes, its penetration of hive space, compatibility with hive substrates (e.g., wax, wood), the process of treatment application (*i.e*., cost and equipment availability) (see, (39)), and the effect of iHP on honey bees. iHP treatment appears to inactivate honey bee microbial pathogens as efficaciously as other tested treatments, yet other tradeoffs remain that could make its use more or less desirable than other treatment types. For example, both iHP (and H_2_O_2_ gas) and ethylene oxide have limited penetration of hive substrates, and water repellency of wax and possibly propolis, make iHP (H_2_O_2_ in aqueous mist) less likely to penetrate inside these substrates. This is in contrast with gamma irradiation, which can penetrate dense materials (22). However, as a gas, iHP can reach hive substrates more readily than can irradiation from a point source, hence the inability to penetrate substrates is less important. Further, both ethylene oxide and gamma irradiation are harmful to users, and at least for the former, can produce biproducts (*e.g*., ethylene glycol) that are toxic to honey bees (40). Finally, iHP treatment appears to have the shortest treatment duration, with high efficacy achieved with only minutes of exposure, compared to hours for gamma irradiation and days for ethylene oxide (see, (22)). Overall, iHP, ethylene oxide, and gamma irradiation show disinfectant properties across a broad range of materials (see, (39)), and decisions for which method is most suitable for the beekeeping industry will depend on cost, availability and safety. Future work could investigate the utility of H_2_O_2_ gas, which may be easier and less costly to apply than iHP vapor, to inactivate honey bee viral pathogens. Both iHP and H_2_O_2_ gas have been tested (the former in limited field trials, the latter in lab only) for their safety to honey bees. In no cases were detrimental effects on honey bees observed (data not shown).

## Data availability statement

The raw data supporting the conclusions of this article will be made available by the authors, without undue reservation.

## Author contributions

SC, CB and ER conceptualized the research. SC, ER, FP and CR conducted the research. SC and ER wrote the manuscript. SC, JE and YC provided funding. All authors contributed to the article and approved the submitted version.
